# Fundamentals of light-cell–polymer interactions in photo-cross-linking based bioprinting

**DOI:** 10.1063/5.0022693

**Published:** 2020-10-12

**Authors:** Daniel Nieto, Juan Antonio Marchal Corrales, Alberto Jorge de Mora, Lorenzo Moroni

**Affiliations:** 1 Photonics4Life Research Group, Department of Applied Physics, Faculty of Physics, University of Santiago de Compostela, Santiago de Compostela 15782, Spain; 2 Complex Tissue Regeneration Department, MERLN Institute for Technology Inspired Regenerative Medicine, Universiteitssingel 40, 6229ER Maastricht, The Netherlands; 3 Department of Human Anatomy and Embryology, Institute of Biopathology and Regenerative Medicine, University of Granada, Granada 18016, Spain; 4 Instituto de Investigación Biosanitaria de Granada (ibs.GRANADA), Granada 18012, Spain; 5 Excellence Research Unit “Modeling Nature” (MNat), University of Granada, Granada 18016, Spain; 6 SERGAS (Galician Health Service) and IDIS (Health Research Institute of Santiago de Compostela (IDIS), Orthopaedic Department, Universidad de Santiago de Compostela, Santiago de Compostela 15782, Spain

## Abstract

Biofabrication technologies that use light for polymerization of biomaterials have made significant progress in the quality, resolution, and generation of precise complex tissue structures. In recent years, the evolution of these technologies has been growing along with the development of new photocurable resins and photoinitiators that are biocompatible and biodegradable with bioactive properties. Such evolution has allowed the progress of a large number of tissue engineering applications. Flexibility in the design, scale, and resolution and wide applicability of technologies are strongly dependent on the understanding of the biophysics involved in the biofabrication process. In particular, understanding cell–light interactions is crucial when bioprinting using cell-laden biomaterials. Here, we summarize some theoretical mechanisms, which condition cell response during bioprinting using light based technologies. We take a brief look at the light–biomaterial interaction for a better understanding of how linear effects (refraction, reflection, absorption, emission, and scattering) and nonlinear effects (two-photon absorption) influence the biofabricated tissue structures and identify the different parameters essential for maintaining cell viability during and after bioprinting.

## I. INTRODUCTION

Bioprinting is a fast emerging technique, which makes biofabricating artificial tissues and the microscale deposition of living cells possible.^1,2^ The ultimate objective of bioprinting is to create 3D artificial tissues that mimic the natural biological microenvironments, where cells can function as well as they would in real tissues. The structural geometry and morphology of artificial structures should be controlled using bioprinting tools to maintain high functionality at various dimension scales to mimic the tissue complexity.^3,4^

Light-based biofabrication methods have been developed and used to generate biological scaffolds and complex tissue structures.^5,6^ The optical nature of light (noncontact, optically selective, and precise processing) positions these technologies at the forefront of biofabrication techniques with the ability for high precision biofabrication at submicrometer resolution (<1 *μ*m).

Most light based printers can be divided by the light source used for polymerization (using one photon or two photons), which is then projected over a bath filled with liquid photo to cross-linkable biomaterials or cell-laden hydrogels onto a moving stage. Most common light-based technologies using photopolymerization include laser-based SLA, mask-based SLA, and digital light projection (DLP) using digital mirror devices (DMDs). These technologies are based on one photon polymerization, initially using UV light^7–10^ and more recently visible light.^11–13^ On the other hand, multiphoton polymerization-based 3D laser lithography^14^ is based on the absorption of two photons of near infrared light (NIR), to excite the same energy transition as ultraviolet (UV)one-photon absorption for cross-linking the biomaterial.

### A. SLA

**FIG. 1.**
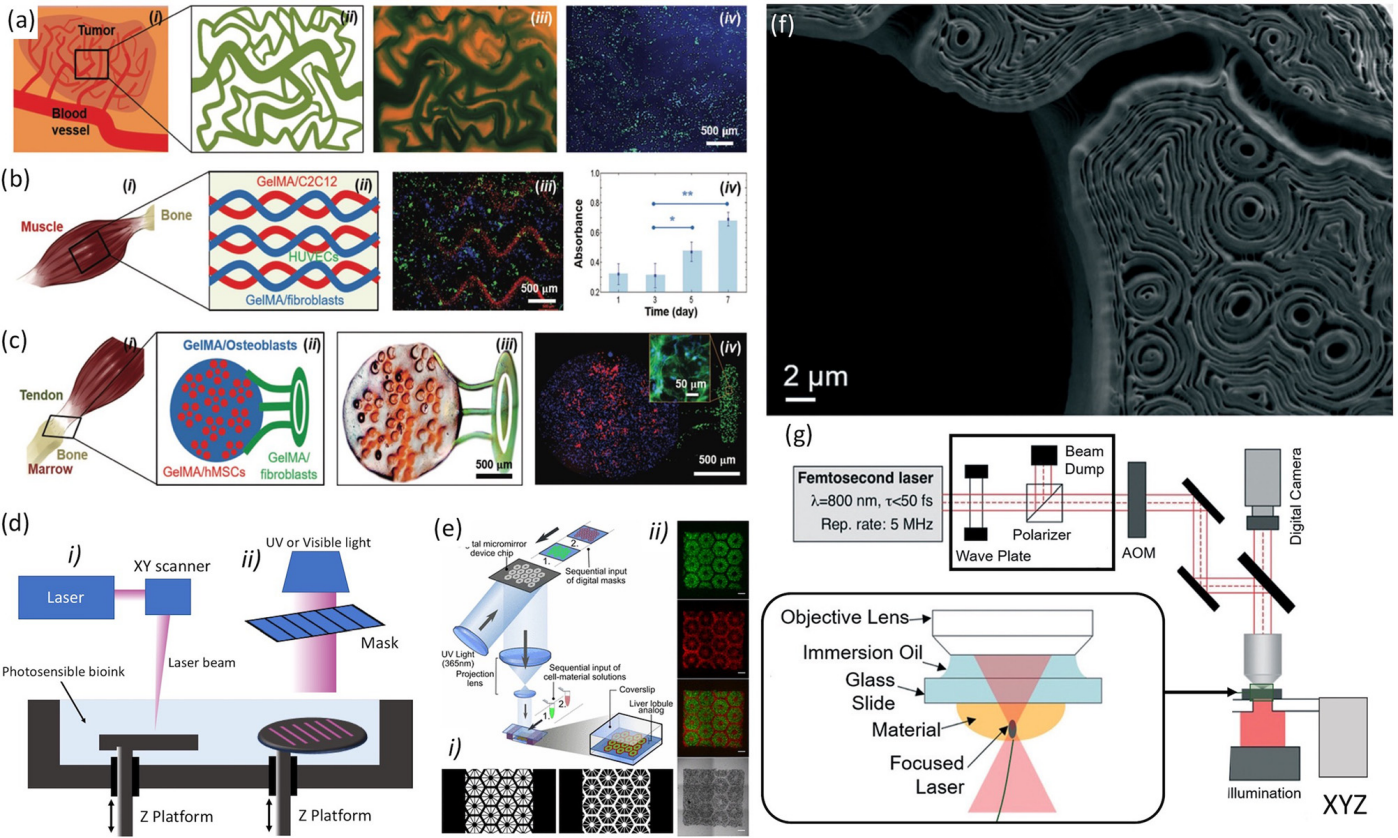
(a) A tumor angiogenesis model: (i) schematic showing the tumor angiogenesis model; (ii) schematic of the mask for printing; (iii) bioprinted microvasculature; and (iv) bioprinted tumor model. (b) A skeletal muscle model: (i) schematic showing the skeletal muscle tissue; (ii) schematic of the mask for printing; (iii) bioprinted skeletal tissue model; and (iv) PrestoBlue measurements of cell proliferation in the bioprinted structures. (c) A tendon-to-bone insertion model: (i) schematic of the tendon-to-bone insertion site; (ii) schematic of the mask for printing; (iii) bright-field optical image showing a bioprinted dye-laden GelMA structure; and (iv) bioprinted tendon-to-bone model. Reproduced with permission from Miri *et al.*, Adv. Mater. **30**, 1800242 (2018). Copyright 2018 John Wiley and Sons. (d) Schematics of stereolithographic bioprinting process: (i) laser-based and (ii) mask-based. (e) Schematic of the DLP bioprinting process: (i) gray scale digital mask and (ii) images of fluorescently labeled hiPSC-derived hepatic progenitor cells (hiPSC-HPCs). Reproduced with permission from Ma *et al.*, Proc. Natl. Acad. Sci. U. S. A. **113**, 2206–2211 (2016). Copyright 2016 PNAS. (f) The cross section of a TPP-bioprinted mouse paw bone imaged using scanning electron microscopy and the intricate contours within the structure that arose from the bioprinting process. (g) Schematic illustration of the experimental setup for two-photon bioprinting along with a zoomed description of focal plane and distribution of light intensity in the laser focus of a Gaussian beam is shown. Images (f) and (g) were reproduced with permission Miri *et al.*, Lab Chip **19**, 2019–2037 (2019).^50^ Copyright 2019 Royal Society of Chemistry.

Conventional SLA was in fact the first technology introduced by Charles Hull in the 1980s for creating 3D constructs using UV light to polymerize materials.^15^ This was followed by other studies discussing the kinetic modeling of linear, cross-linking photopolymerization and highlighting the opportunities as industrially cured coatings and dental fillings, and more generally three-dimensional rapid prototyping techniques.^16,17^ SLA bioprinting presents some advantages in comparison with other bioprinting techniques, such as extrusion and inkjet systems. SLA bioprints photosensitive hydrogels in a layer by layer fashion rather than in struts or droplets. The bioprinting time for each layer is the same, but the total biofabrication time of the hole structure depends on the thickness. This bioprinting characteristic of SLA reduces the biofabrication time of the hole tissue structure. Moreover, SLA is a nozzle-free bioprinting technique, which avoids the inconvenience associated with nozzle-based bioprinting technologies, resulting in cell-laden structures with viability higher than 90%.^18,19^ Commonly, SLA uses the light source that impinges into a bath filled with a photosensitive biomaterial, which is placed onto a Z moving stage. The Z stage moves down to a predefined distance and the material is polymerized. This distance determines the vertical resolution of the SLA printer. More recently, a top-down approach was used, where the light source that was placed below the bath containing the biomaterial, in this case, the Z platform, is moved up to a distance which determines the thickness of the layer and hence Z resolution. Mask-based SLA uses a mask and a light source to project the photomask [Fig. 1(d)]. Laser-based SLA uses laser, which is focused using a lens with XYZ movement to transfer the pattern. Bioprinting speeds using SLA are higher than other conventional methods. Nevertheless, this method presented poor biocompatibility with low resolution.^20^ Although, initial studies have reported feature sixes of 150 *μ*m per single layer and with axial resolution ∼250 *μ*m,^21,22^ with the development of biomaterials and hydrogels, the biocompatibility and resolution of SLA improved to 50 *μ*m.^18^ SLAs have been commonly used in manufacturing industries and more recently used for tissue engineering applications. Initially, SLA has been used for bone tissue models. Catros *et al.* used an SLA bioprinter for patterning nanohydroxyapatite (nHA) and osteoblastic cells in 2D and adapted to the biofabrication of 3D composite materials toward healing bone defects.^23^ Wang used an SLA bioprinting system in combination with visible photosensitive bioinks [poly(ethylene glycol) diacrylate (PEGDA), gelatin methacryloyl (GelMA), and eosin Y based photoinitiator], resulting in NIH 3T3 cell bioprinting with 50 *μ*m resolution and high cell viability.^24^

### B. Digital light projection

The printing speed of SLA can be significantly improved by using mask-less DMD-based bioprinting. The DMD-based bioprinting uses an array of micromirrors (the dimension of each micromirror can be in the order of 5–10 *μ*m) to selectively switch the light intensity of each micromirror (each individual mirror can be controlled on two positions, being either 0-dark or 1-light reflecting and with speeds on the order of kilohertz) and project it over light-sensitive biopolymers that polymerize the preselected light patterns transferred by the DMD in a layer by layer fashion [Fig. 1(e)]. DMD technology has arisen as an alternative for high-throughput DLP printing, resulting in good biocompatibility for seeding cells.^25^ Zhu *et al.* used a DMD bioprinter for generating prevascularized tissue models with complex geometries (widths ≤50 *μ*m and heights ≅50 *μ*m) using a bioink of endothelial cells, GelMA, and glycidal methacrylate–hyaluronic acid.^26^ Miri *et al.* were able to generate biological tissue structures such as tumor angiogenesis [Fig. 1(a)], muscle strips [Fig. 1(b)], and musculoskeletal junctions [Fig. 1(c)] with printing resolutions on the order of 10 *μ*m by using a DMD bioprinter working at 365 nm, in combination with microfluidics.^8^ Ma *et al.* have used DMD to fabricate hexagonal lobule structures of GelMA (15% w/v) seeded with HUVECs (Human Umbilical Vein Endothelial Cells) (with a resolution ∼50 *μ*m) that where incorporated on a liver-on-a-chip.^27^ These studies highlight the high speed of bioprinting associated with DMD (under 1 min), accuracy (10–50 *μ*m), and versatility (from biocompatible scaffolds to cell-laden structures with different geometries) of the mask-less methods.

### C. Multiphoton polymerization-based 3D laser lithography

The challenge associated with 3D biofabrication using single-photon photopolymerization is to avoid the off-focal photopolymerization that may ultimately cure undesirable parts of the designed construct.^28,29^ Biofabrication using multiphoton polymerization benefits from the high resolution inherent in the two-photon polymerization (TPP) process, which can generate 3D structures with micro/nanoscale resolution [Fig. 1(f)].^30^ The nonlinear optical phenomenon associated with TPP occurs when irradiating using a focused femtosecond laser beam at infrared wavelength, by simultaneous absorption of multiple photons, which induces photopolymerization of a small area (∼100 nm), based on the radical generation due to the interaction between the used photoinitiator and the femtosecond laser beam. This interaction allows the generation of 3D tissue structures with ultra-high-resolution (from *μ*m to nm) that cannot be achieved by other conventional photolithographic methods.^31^ Ovsianikov *et al.* used TPP to fabricate biodegradable tissue scaffolds using gelatin modified with methacrylamide (GelMod), which were seeding with adipose-derived stem cells, presenting good adhesion and resulting in proliferation and differentiation to adipocytes.^32^ Koroleva *et al.* demonstrated that hybrid Zr–Si porous scaffolds were fabricated using TPP promoted mesenchymal stem cells (hMSCs) to differentiate toward the osteogenic lineage.^33^ Commonly used TPP systems include two X–Y galvanometric scanners to move the laser focus in the X–Y coordinates and a high resolution Z stage that performs axial scanning [Fig. 1(g)]. Due to the coherent properties of the laser beam with optimal focusing capabilities, the TPP process can generate high-resolution 3D features. Nevertheless, the throughput is restricted by the sequential laser scanning process. This limitation is further enhanced when printing complex hollow structures or large volume structures. Different optical solutions have been proposed, which include microlens arrays, spatial light modulators, and diffractive optical elements, most of them based on splitting the laser into multiple foci. Geng *et al.* have used TPP in combination with a DMD scanner for generating tens of laser foci that can be controlled individually by achieving diffraction-limited resolution (500 nm–1600 nm) and a processing speed of 22.7 kHz.^34^ These studies highlighted the opportunities of TPP associated with high resolution features. Nevertheless, several key challenges still remain, which include the failure of biofabricating cell-laden constructs with clinically relevant dimensions. TPP systems that are commercially available are very expensive and are difficult to adapt to the particular application. The dearth of biomaterials (biocompatible and biodegradables) for TPP is another inconvenience for covering different biological applications. The dearth of water soluble PIs limits the uses of photopolymers with high water contents. Although TPP is very precise, it is a relatively slow process, which results in small scaffolds of structures difficult to handle in tissue engineering. Biological experiments need statistical experiments with a huge amount of identical structures. In this sense, efforts need to be taken to develop novel photopolymers and photoinitiators for TPP aimed at increasing the fabrication speed and reducing cytotoxic effects.

Table I shows the characteristics of the most common light based technologies using photopolymerization.

**TABLE I.** Most common light based bioprinting technologies using photopolymerization.

Bioprinting technology	Advantages	Common advantages	Disadvantages	Common disadvantages	References
Digital light projection (DMD)	High cell viability Direct incorporation of cells during bioprinting Dynamic bioprinting High resolution	Noncontact biofabrication systems (No shear, mechanical and thermal stress, nor clogging during bioprinting) High resolution and density; additive operation Photopolymerization is cell friendly (pH, temperature)	Customized systems/required skills Moderate cost for high resolution systems UV light can damage micromirrors	Require photocurable bioink (limited biomaterials) Monomer toxicity (biomaterial reactions during bioprinting) Custom made equipment (require technical staff)	8 9 27
Laser-based SLA	High resolution (1 − 50 *μ*m) Bioprinting of high viscosity Selective exposure of bioinks		Medium speed Limited scalability Laser source might have an adverse effect on the cellular genetic material Moderate cost for high resolution systems		6 12 27
Mask-based SLA	High cell viability Easy control of matrix properties Low cost technology Fast speed		Monomer toxicity and use of ultraviolet radiation Require a mask pattern Multiple step processes		3 25 27
Multiphoton bioprinting	Ultra-high resolution (nm to few micrometers) High penetration depth No UV light required High water content bioinks		Limited by the speed of printing for high-throughput screening High cost technology Require optimization of photocurable bioink		11 24 25 27

### D. Basic light interaction process during bioprinting

During photolithographic biofabrication, the primary light interactions can be caused mainly by linear phenomena of refraction, reflection, absorption, emission, and scattering and nonlinear effects as in the case of multiphoton polymerization.^35^ Reflection occurs when light reflects on the surface of the biomaterial without penetrating it. Refraction occurs as a consequence of the change in the propagation angle of light when it passes from air to the biomaterial during bioprinting. Absorption and dispersion that occur both in a biological biomaterial and in cells are dependent on the wavelength of light, which is complete through intracellular and extracellular constituents. Emission is a phenomenon that consists of an energy transmission to the atoms of the biological component, in which their electrons are promoted to higher levels of energy. While the energy of photons used to irradiate cells has a dramatic impact on phototoxicity, it can be expected that the bioprinting limitations are mostly related to the photon energy and the wavelength of the light source used to photo-cross-link the biomaterials. Although cells exhibit a very distinct irradiation sensitivity, the phototoxicity increases dramatically with decreasing irradiation wavelength.^36^ Infrared light, as long as we do not pass the cellular thermal threshold through which apoptosis and destruction are induced, is safe. Nevertheless, ultraviolet light is mostly used for light-curing photopolymerization on stereolithographic bioprinting and has been reported to damage the DNA of cells.^37,38^

Following light absorption, in certain circumstances, cells can undergo a wide variety of photochemical and photophysical processes, which include fluorescence, thermal effects, photoablation effects, plasma-induced ablation, and photodisruption.^39^ These effects must be avoided during bioprinting. Photoablation occurs due to the action of an intense ultraviolet (UV) laser pulse that photochemically decomposes various cellular and extracellular components. Plasma-induced ablation and photodisruption occur as a consequence of exposing the biological material to a power density above 1011 W/cm^2^.^40^ Fluorescence originates from the transition from an excited singlet state to a ground state vibrational mode. Thermal effects are the result of the conversion of absorbed light energy into heat. The above-mentioned mechanisms need to be considered carefully to increase cell viability. Light power, selected wavelengths, and exposure time are perhaps the main determinants that dominate the process during light based biofabrication. It is essential to adapt the light beam and optimize the light parameter to minimize cell damage without losing its ability to light cure or achieve cross-linking in the medium. Most Stereolithography (SLA) printers can be divided by the light source used for polymerization (using one photon or two photons), which is then projected over a bath filled with liquid photo to cross-linkable biomaterials or cell-laden hydrogels onto a moving stage. Although photo-cross-linking is typically associated with SLA bioprinting, other biofabrication technologies, such as extrusion, may use photo-cross-linking as a secondary process. Bram *et al.* have used extrusion bioprinting based on a two-step cross-linking approach. Secondary photo-cross-linking was applied for shape maintenance. This two-step cross-linking methodology can be used with a broad window of extrusion biofabrication parameters that allow printing at a low viscosity (4 mPa s) to maintain high cell viability (>80%) and with good shape fidelity. This eliminates the problems associated with low viscosity bioinks (complex chemical modifications, multiple initiation systems, and viscosity enhancers).^59^ There is a vast amount of reviews covering the advantage and disadvantages of different bioprinting technologies and more recently the fundamentals and practical aspects of light based bioprinting,^41,42^ but few of them covering the primary cell–light interactions.

In this article, we consider the impact and behavior of light during bioprinting, focusing on high resolution structures with high cell viability. Section II is related to polymer–light interactions centered on high resolution structures. Section III is devoted to biophysical principles at that cell–light interaction level and parameters involved to maintain high cell viability, and Sec. IV presents a future outlook and conclusions.

## II. POLYMER–LIGHT INTERACTIONS

Photopolymerization comprises the reaction of monomers that form large networks when irradiated with light (by the single-photon or two-photon absorption). This absorption can be promoted by the reactant monomer or by the transfer of energy absorbed by a photoinitiator.^43^ Photoinitiators used for photopolymerization generate free radicals when they are exposed to light and react with monomers and/or oligomers for initiating polymer chain reactions and growth.

### A. Photopolymerization mechanism (single-photon vs two-photon)

**FIG. 2.**
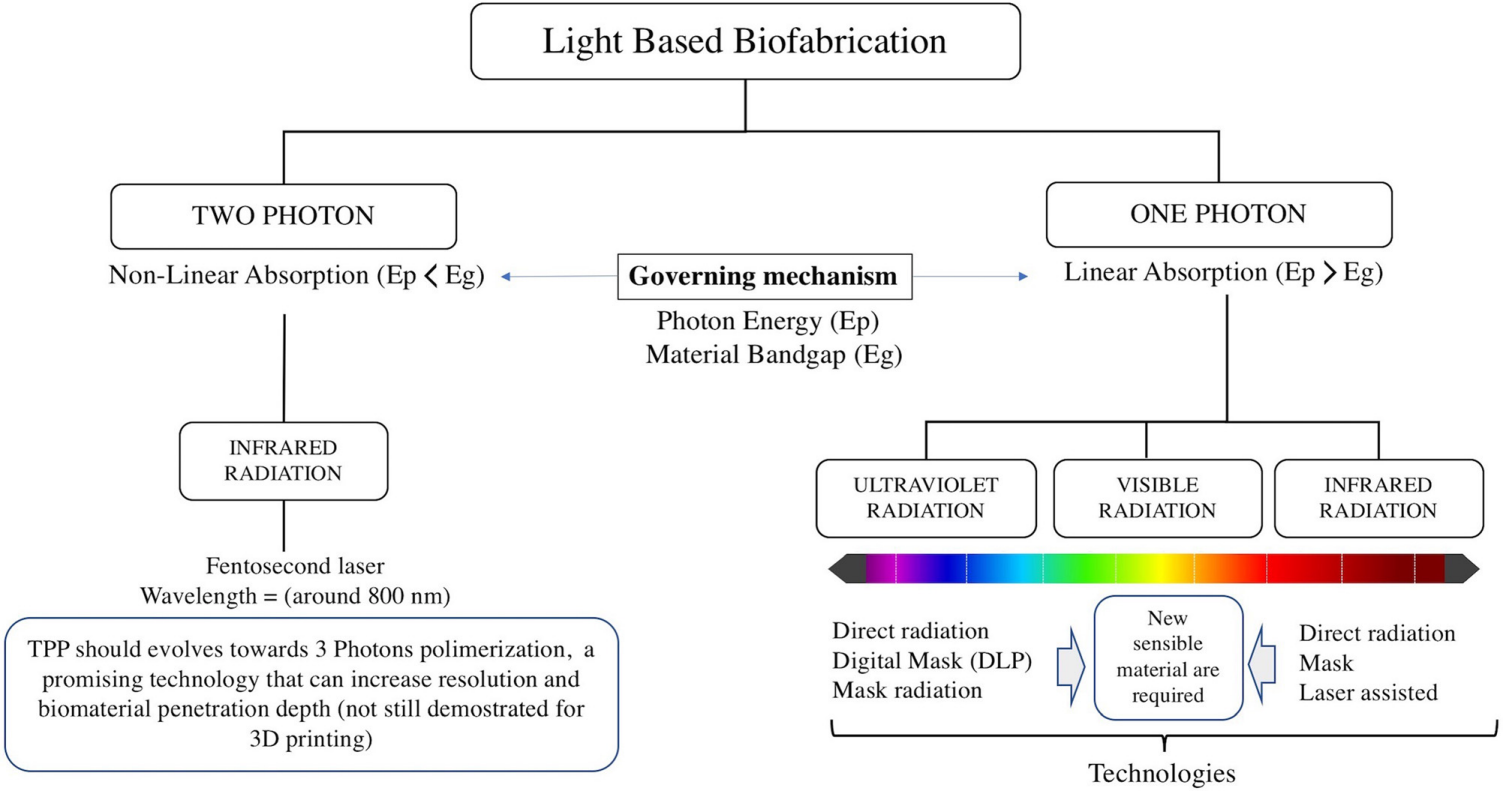
Diagram of stereolithographic processes based on the exciton radiation form and energy (single photon and multi-photon).

During photopolymerization, the photon interactions at the initial step differ from the ordinary thermal polymerization, but the following steps being propagation, termination, and chain transfer remain the same. Under such premises, the photopolymerization bioprinting process can be classified into two categories: single-photon photopolymerization and multiphoton photopolymerization (Fig. 2).

**FIG. 3.**
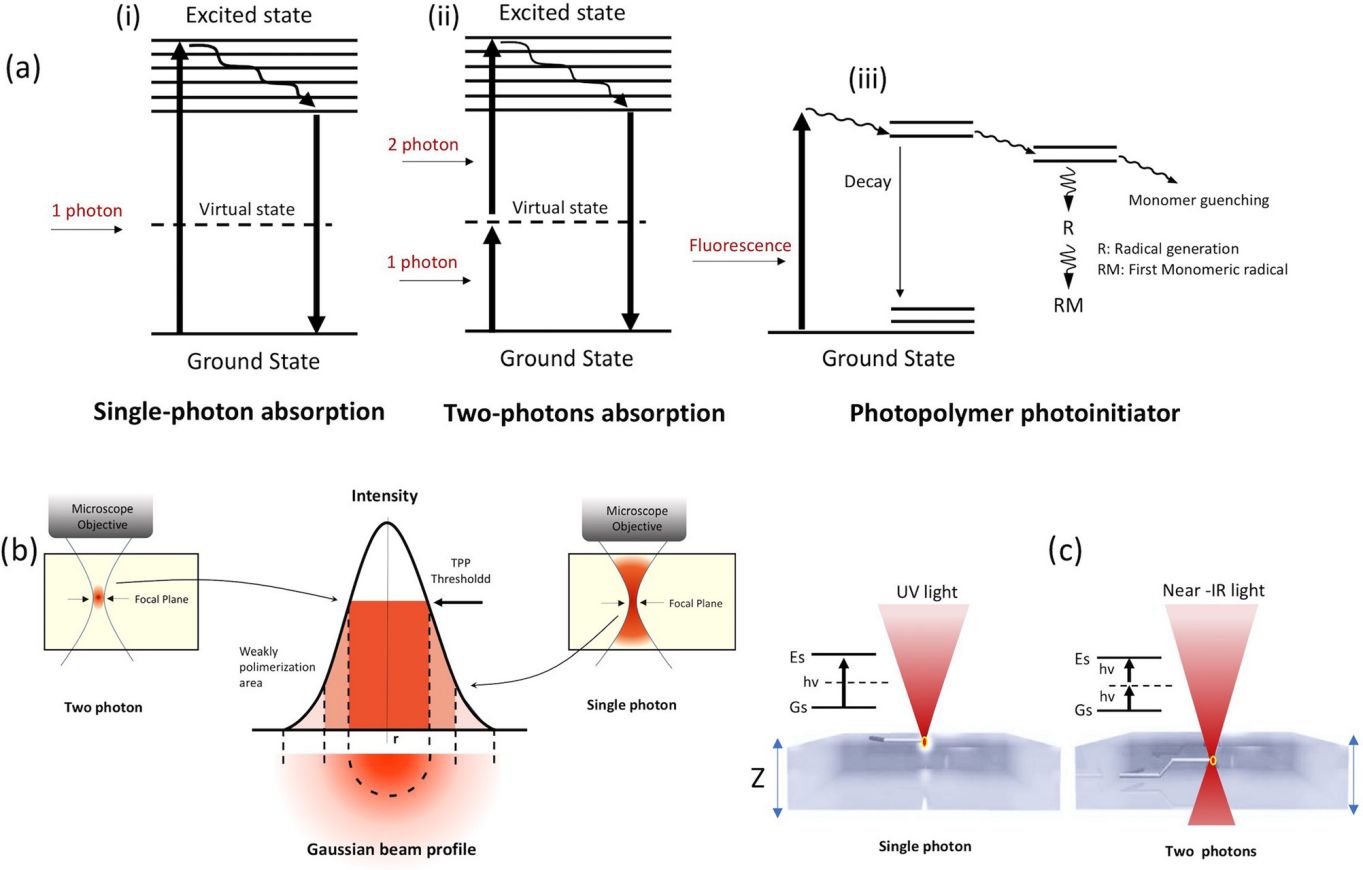
(a) Single-photon and two-photon absorption processes, (b) Gaussian beam profile of a laser beam, and (c) single-photon and two photon absorption features on the biomaterial.

In single-photon SLA, the polymerization process is originated via linear single-photon absorption [Fig. 3(a)].^44^ The energy of the photon, E_p_, is equivalent or superior than the material bandgap E_g_. With $E_{p}=hv=\mathrm{hc}/\lambda$ being the governing law of this process, it means that high energy photons and short wavelengths are required. In most cases, UV wavelengths shorter than 365 nm are selected. One photon absorption with $E_{p}>E_{g}$ motivates the electron to move from the valence band to the conduction band. This process alters the chemical bond inducing polymerization process, which is responsible for biomaterial cross-linking. Noncoherent light sources at low power levels can also induce linear absorption. Nevertheless, as the light intensity increases, typically using coherence laser sources, nonlinear absorption can take place. The UV (λ_UV_) photosensible biomaterial can be also photopolymerized by infrared (IR) wavelengths of nearly double wavelength (λ_IR_ = 2λ_UV_).^45^ TPP is based on this mechanism and comprises the absorption of two photons simultaneously through a virtual state for molecule excitation [Fig. 3(a)]. The virtual levels (∼fs) have a particularly short lifetime, which results in the instantaneous absorption of almost two photons. This excitation process depends quadratically on the incident light intensity^46^ and the TPP requires high light intensities (>GW/cm^2^). The TPP process can be described as follows:^47^ a molecule in a ground energy state Gs is excited to an excited state Es. In this process, the molecules absorb two single photons (Gs and Es are separated with an energy difference of Ef above the Gs) as shown in Fig. 3(a). A virtual state Vs is created by the absorption of the two photons [both photons having the same energy levels = E1 (degenerate, Ef = 2E1)]. With further excitation, they lose energy and move to another state R, where vibrational relaxation is induced by the lowest vibrational level of the lowest-energy Es [Fig. 3(a), dashed arrow], and then return to the ground state by a pathway that can be radiative or nonradiative.

The TPP process is originated precisely at the focal volume of the laser beam [Fig. 3(b)], which facilitates the generation of precise and high-resolution 3D structures. The biggest limiting factor in the extensive use of TPP for biological applications is the slow manufacturing time that accompanies high-resolution structuring which can compromise cell viability. This can be overcome by using optical systems to modulate the behavior of light. Gittard *et al.* have demonstrated TPP using a multiple spotlight approach by generating microstructure arrays for tissue engineering. Computer-generated hologram patterns were used to generate multiple spotlights from one laser beam, significantly reducing the manufacturing time. These multiple foci were used to simultaneously produce multiple tissue scaffolds by TPP.^48^ Atry *et al.* demonstrated the applicability of diffractive optical elements for fabricating large scaffolds at rates several times faster than by single spotlight.^49^

The two key properties that conditioned the resolution of laser direct write bioprinting, mask based SLA, and DMD-based bioprinting processes, which are mainly determined by the thickness of the photosensitive resin, are the directionality of the impinging light and the lower scattering of light (perpendicular to the laser). The thickness (z resolution) can be controlled by adjusting the laser characteristics (pulse width, light wavelength, power, repetition rate, and size of the beam) and the properties of the resin (including viscosity and superficial stress).^50^ According to the polymerization kinetics of the photo-cross-linking mechanisms, we can assume the following relationship to define the thickness of the light-cured material:

$$C_{d}=D_{p}\text{ln}\left[\frac{E_{i}}{E_{c}}\right],$$

where C_d_ is the depth of curing (*μ*m), D_p_ is the depth of penetration (*μ*m), E_i_ is the irradiation of light (mJ/cm^2^), and E_c_ is the threshold value of energy of the gelation point for the liquid resin (mJ/cm^2^). As E_i_ approaches E_c_, the layer is cured, and the resin is solidified. Because of the nonlinear nature of two photon polymerization and the threshold behavior, high-quality and high-resolution features can be obtained. By adjusting different laser parameters (pulse energy and pulse repetition rate), the printing resolution (<100 nm) can be increased by overcoming the diffraction limit.

The two-photon absorption mechanism happens in a resin that initially does not absorb the selected wavelength of the laser light, allowing its penetration in the material [Fig. 3(c)]. The bioprinting resolution of TPP is related to the incident laser light and the square of its intensity. A high magnification focal lens focuses laser energy at a small focal point where the highest amount of absorption takes place. Assuming a Gaussian laser beam profile with an intensity distribution I(r, z) at distances (z in the direction of propagation and r along the cross section) from the center can be defined as

$$I\left(r,z\right)=I_{0}\left[\frac{w_{0}^{2}}{{w(z)}^{2}}\right]e^{\frac{{-2r}^{2}}{w{\left(z\right)}^{2}}},$$

where I_0_, ω_0_, and ω(z) are the intensity at the center of the Gaussian beam (r = 0, z = 0), the waist of the beam, and the radius of the beam in the plane with a distance of z, respectively. The average intensity at the focus plane can be defined as

$$I_{\mathrm{focus}}=\frac{w}{\pi \;{w_{0}}^{2}\;\varsigma \;fh\upsilon },$$

where W is the power average, ς is the width of the selected pulse, f is the repetition rate, h is the Planck constant, and υ is the frequency of light. The photon-polymerization is initiated when the density of radicals P (r, z) surpasses the threshold Pth [P (r, z) ≥ Pth]. The intensity at the focal plane (z = 0) reaches the threshold, where

$$I\left(r,z\right)=I\left(r,0\right)=I_{0}\;\exp\left(\frac{{-2r}^{2}}{{w_{0}}^{2}}\right).$$

All the aforementioned interactions and effects have influence over the bioprinting process.

### B. Photoinitiators

The polymerization efficiency of the developed bioinks depends strongly on the selection of the photoinitiator. The photoinitiator should be efficient in free radical generation with low toxicity. For engineering of living tissues, photoinitiators sensitive to UV are the most used. Photoinitiators can be separated into two categories (in relationship with the radical generation mechanisms): (1) Type-I photoinitiators (cleavable photoinitiators) and (2) Type-II (bimolecular photoinitiating). During bioprinting, for initiating polymerization, Type-I photoinitiators generate two radicals. The starting process of Type-II (e.g., benzophenone/tertiary amine) presents more complexity. For example, benzophenone is excited and promotes fast electron transfer (from the lone pair of tertiary amine), which is followed by the proton transfer process; this process provides the radical (H-donor) that initiates photopolymerization. To avoid the UV light damaging effects, including DNA damage and cancer effects,^51^ some visible light photoinitiators have been investigated and demonstrated to be useful for bioprinting with cells. LAP (lithium phenyl-2,4,6-trimethylbenzoylphosphinate) is a UV photosensible photoinitiator, which has also been demonstrated to be sensitive to blue light (near UV).^52^ Eosin Y (2′,4′,5′,7′-tetrabromofluorescein disodium salt) is sensitive around 514 nm. Hydrogels developed for working with Eosin Y maintain cell function and present less toxicity than Irgacure 2959 (1-[4-(2-hydroxyethoxy)-phenyl]-2-hydroxy-2-methyl-1-propanone).^53–55^ Natural photoinitiators such as Riboflavin (FR) and Vitamin B2, were demonstrated to induce photo-cross-linking on alginate hydrogels^56^ and were used as visible light photoinitiators in the thiol–ene polymerization of polyethylene glycol (PEG)-based hydrogels.^57^ A photoinitiator based on a ruthenium complex [tris-bipyridyl-ruthenium (II) hexahydrate] and sodium persulfate (SPS) was used to initiate visible light cross-linking of hyaluronic acid/gelatin-based bioinks.^58^ Soliman *et al.* have used ruthenium (Ru) and sodium persulfate (SPS) cross-linkers in combination with allyl‐functionalized gelatin (Gel‐AGE) bioink for extrusion bioprinting based on the dual‐step cross-linking approach, where a primary (partial) cross-linking in the absence of light is performed to alter the bioink's rheological properties with subsequent secondary post‐printing cross-linking for shape maintenance.^59^ Lim *et al.* have used a Vis + Ru/SPS system, demonstrating better cell cytocompatibility than the commonly used UV + I2959 system. Encapsulated cells remained >85% viable even when using high Ru/SPS concentrations, visible-light intensities, and longtime exposure times (21 days), which highlight the potential Vis + Ru/SPS system to avoid the cell damage associated with UV light and for maintaining high cell viability, shape fidelity, and metabolic activity.^60^ More recently, poly-α-ketoester based photoinitiators have demonstrated good cell viability in combination with methacrylates and polyethylene glycol (PEG) diacrylate-based hydrogels.^61^ It appears, therefore, clear that visible light sensible materials are emerging as optimal PI for cell-laden bioinks for bioprinting. A list of the commonly used PIs and their light absorbing peaks are showed in Table II.

**TABLE II.** Common PIs used in light-based bioprinting.

Name (chemical)	Abbreviation	Absorbing peak (nm)	Sources
2′,4′,5′,7′-Tetrabromofluorescein disodium salt	Eosin Y	514	54, 55
2,2′-Azobis[2-methyl-n-(2-hydroxyethyl)propionamide]	VA-086	385	53
Lithium phenyl-2,4,6-trimethylbenzoylphosphinate	LAP	375	52
1-[4–(2-Hydroxyethoxy)-phenyl]-2-hydroxy-2-methyl-1-propanone	Irgacure 2959	257	8
Riboflavin (Vitamin B2)	RF	220–240	56, 57
Ruthenium with a reagent (sodium persulfate)	Ru (SPS)	400–450	58–60
Poly-α-ketoester based photoinitiators	Poly-α-ketoesters	330	61

## III. CELL–LIGHT INTERACTIONS

Human cells vary in size in a range from 5 *μ*m of erythrocytes (red blood cells), 20 *μ*m of leukocytes, to tens of centimeters of neuronal axons.^62^ They can therefore be either larger or smaller than the light wavelength used for bioprinting. The interaction of cells and cell-laden biomaterials with light can be caused mainly by linear phenomena of refraction, reflection, absorption, emission, and scattering.^63^

During bioprinting, the light refracts when it travels from the light source (by air at a particular angle) and reaches a substance (biomaterial) which presents another refractive index. The refractive index determines the phase and speed of light propagation. Reflection occurs when light reflects on the surface of the biomaterial without penetrating in it, due to the difference between the refractive index of air and biomaterial. Reflection at the microscopic level will depend on the cell surface morphology and because of it will not be uniform. Light absorption is complete through intracellular and extracellular constituents and occurs as a consequence of the transition from a grown state (low energy state) to an excited state (high energy state) of a molecule.^64,65^

Emission is a phenomenon that consists of energy transmission to the atoms of the biological component, in which their electrons are promoted to higher levels of energy, in some circumstances unstable, leaving holes under them. When electrons return to these holes with less energy, the excess of energy is returned as light, with different characteristics from the incident ray. Scattering occurs due to loss of directionality of light and spread of the light beam spot. This phenomenon is what regulates the light intensity distribution in the cell-laden biomaterials.

Light scattering in tissues is dominated by Mie scattering. This type of scattering occurs when particles are the same size as the wavelength of light (when particles are much smaller than the wavelength, Rayleigh scattering predominates). Cells, nuclei, and organelles all fall into this classification. In addition, the lipid membranes that enclose these structures have a different refractive index than the surrounding medium (around 1.5). The dispersion of light into the tissue is motivated by the differences of the refractive index. When the difference between the medium and the cells increases, the dispersion also increases. The relationship with the wavelength can be complex, but in general, the longer the wavelength, the lower the scattering, which indeed is one of the benefits of TPP.^66^

**FIG. 4.**
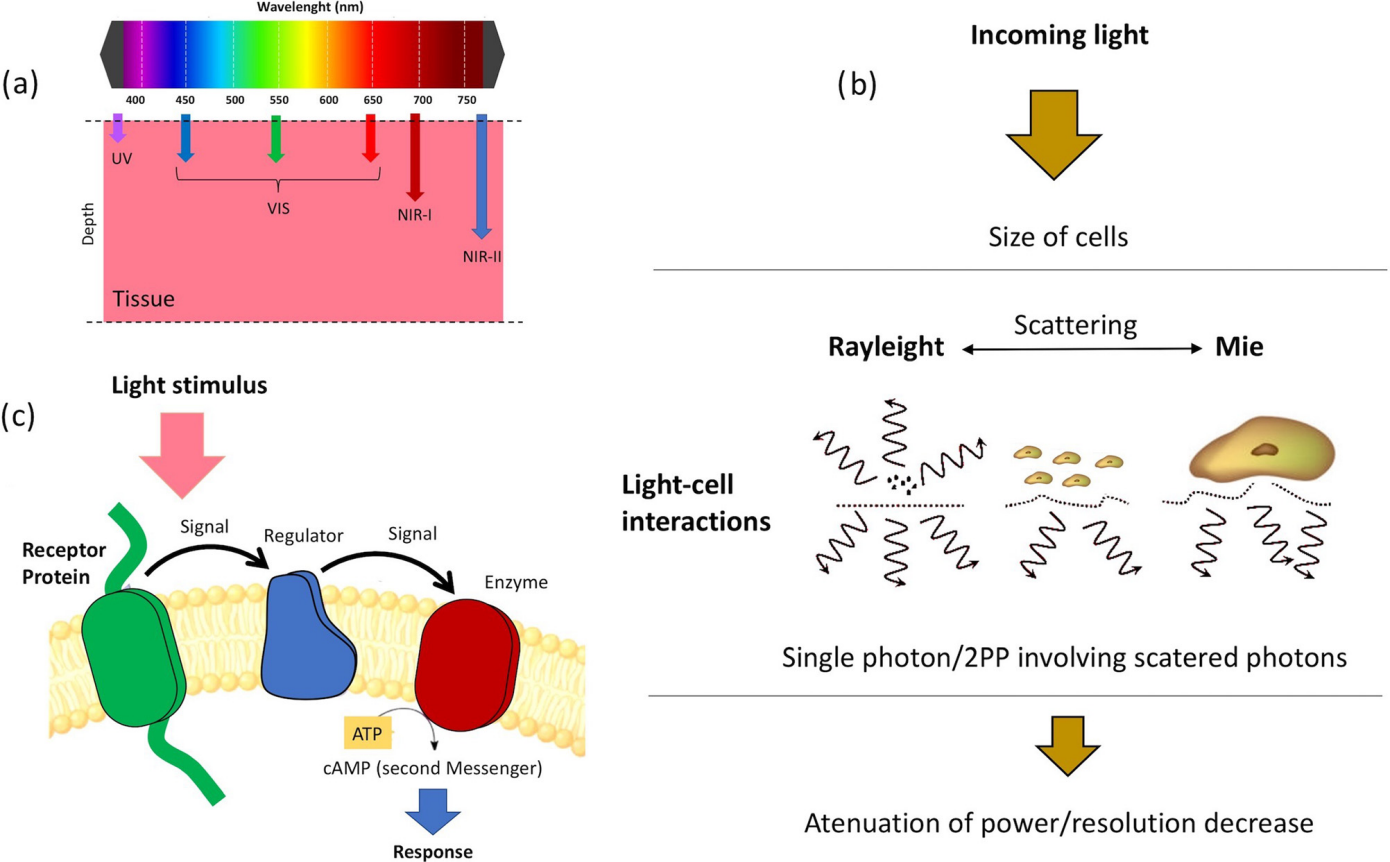
(a) Wavelength dependence of light penetration in cell-laden biomaterials, (b) sketch of light–cell interaction mechanism, and (c) diagram of the cell-laden biomaterial absorption mechanism.

In Rayleigh scattering, subcellular components such as organelles can be a scattering component. This type of scattering depends mostly on the following parameters: the dimensions of the scattering compounds (cells or organelles in bioprinting), scattering centers and the surrounding medium refractive index variations, and the light wavelength [see Fig. 4(b)].^67^ We can consider that Rayleigh scattering is inversely proportional to the square of the wavelength of light. That means that a short wavelength (UV) will be more scattered than a long wavelength (IR). By taking scattering individually as a mechanism for optical loss during bioprinting, we can assume that the longer the wavelength is, the deeper will the light penetrate into a cell laden biomaterial sample [Fig. 4(a)].

Absorption is controlled by Beer's law, especially when working with monochromatic beams.^68^ It establishes empirically an absorption coefficient in the matter, and corelates this absorption to the wavelength of the incident light. In studies mainly on human skin tissues, due to a high interest in being an area permanently exposed to radiation, an increase in absorption and less penetration of light with shorter wavelengths (in studies with wavelengths from 300 to 800 nm) has been demonstrated.^69^ This law is valid in liquid media such as cellular and intracellular interstitial fluids, and establishes an increase in absorption with the concentration of solute in the medium in cell-laden biomaterials. Let us consider a sample which is in a solution, contained in a box which is transparent to the radiation of interest (monochromatic) and with uniform thickness. With *I*_0_ being the intensity of the radiation that enters the sample and *I* being the intensity of the radiation that goes across the sample, the transmittance T is given by T = I/I_0_. Beer's law can be expressed as

$$\log_{10}\left(\frac{I}{I_{0}}\right)=\mathrm{abc},$$

where *b* is the thickness of the box, *c* is the concentration of the sample in the solution, and *a* is the capacity of the sample to absorb radiation. Beer's law can be simplified as A = abc, with A being the absorbance, and is expressed as

$${A=\log}_{10}\left(\frac{I}{I_{0}}\right).$$

Beer's law says that the concentration and the absorbance are linearly proportional (when the cell thickness and the radiation wavelength remain constant). Therefore, both the absorption and the dispersion that occur both in a biological biomaterial and in cells are conditioned by the wavelength and increased in the blue region of the electromagnetic spectrum compared to the red and infrared regions.^69^ Following light absorption, cells undergo a wide variety of photochemical and photophysical processes. Some cellular elements generate fluorescence (emissivity) as they are excited directly or when they get energy from another cellular element. This is defined as autofluorescence and the constituent it emits is called fluorochrome. Fluorescence, which has a half-life between 1 and 10 ns, originates from the energy transition (excited singlet state to a ground state vibrational mode).^70^ Other processes that can be observed in light–biological matter interactions, apart from the autofluorescence discussed above and photochemical processes, are thermal effects, photoablation effects, photodisruption, and plasma-induced ablation.^40^

Thermal effects can be considered as the result of the conversion of absorbed light energy into heat. They can be produced by pulsed and continuous wave (CW) lamps and lasers. They are nonspecific, which means that these effects are not wavelength dependent. Two critical limitations to consider are the maximum level of temperature that the cells can reach and the propagation of heat in the cells. Photoablation is a process that occurs due to the effect of a high intense ultraviolet (UV) light (typically a short laser pulse) that photochemically decomposes cells and extracellular constituents. The produced ablation is tightly localized at the point of the light beam, outside of which it does not occur. The power densities are on the order of 10^7^–10^10^ W/cm^2^. Plasma-induced ablation and photodisruption occur as a consequence of exposing the biological material to an irradiance above 10^11^ W/cm^2^, producing an electric field that causes dielectric breakdown of matter and generates a high electronic density (plasma). The generated plasma absorbs strongly in the UV, visible, and infrared regions causing ablation. With high plasma energies, the structure of the biological material is broken by mechanical impact. Photodisruption is not localized and can spread in biological materials or cells adjacent to the rupture zone.

Infrared light, as long as we do not pass the cellular thermal threshold through which apoptosis and destruction are induced, is safe. Nevertheless, the UV light is mostly used for light-curing photopolymerization on stereolithographic bioprinting. This radiation is the one that is being used with more intensity in new technological bioprinting developments. There are several manufacturers that use wavelengths of approximately 405 nm in 3D photopolymerization bioprinting for the generation of solid structures from cell-laden biomaterials. At the biological level, excessive exposure can induce changes in DNA and is associated with mutagenesis and the appearance of tumors. Cells have mechanisms to fight against light-induced damage (both thermal and mutagenic). Among these mechanisms, we can highlight the activation of molecules of the heat shock protein (HSP) group,^71^ proteins of the p53 group, and anti-inflammatory cascades mediated by IL-17F among others.^51^ Alterations in these signaling pathways or molecules can trigger individual cellular susceptibility to UV radiation [Fig. 3(c)]. This damage can also be cumulative, especially in predisposing cells. Toxicity of UV radiation varied with wavelengths and exposure doses. Masuma *et al.* have investigated the toxicological effects of a range of UV wavelengths (250 nm–310 nm).^72^ The energy doses leading to cell death increased by increasing the wavelength. The lethal dose for killing cells at 250 nm was 120 mJ/cm^2^, while the doses required to damage cells working at 310 nm was 6 J/cm^2^.

**FIG. 5.**
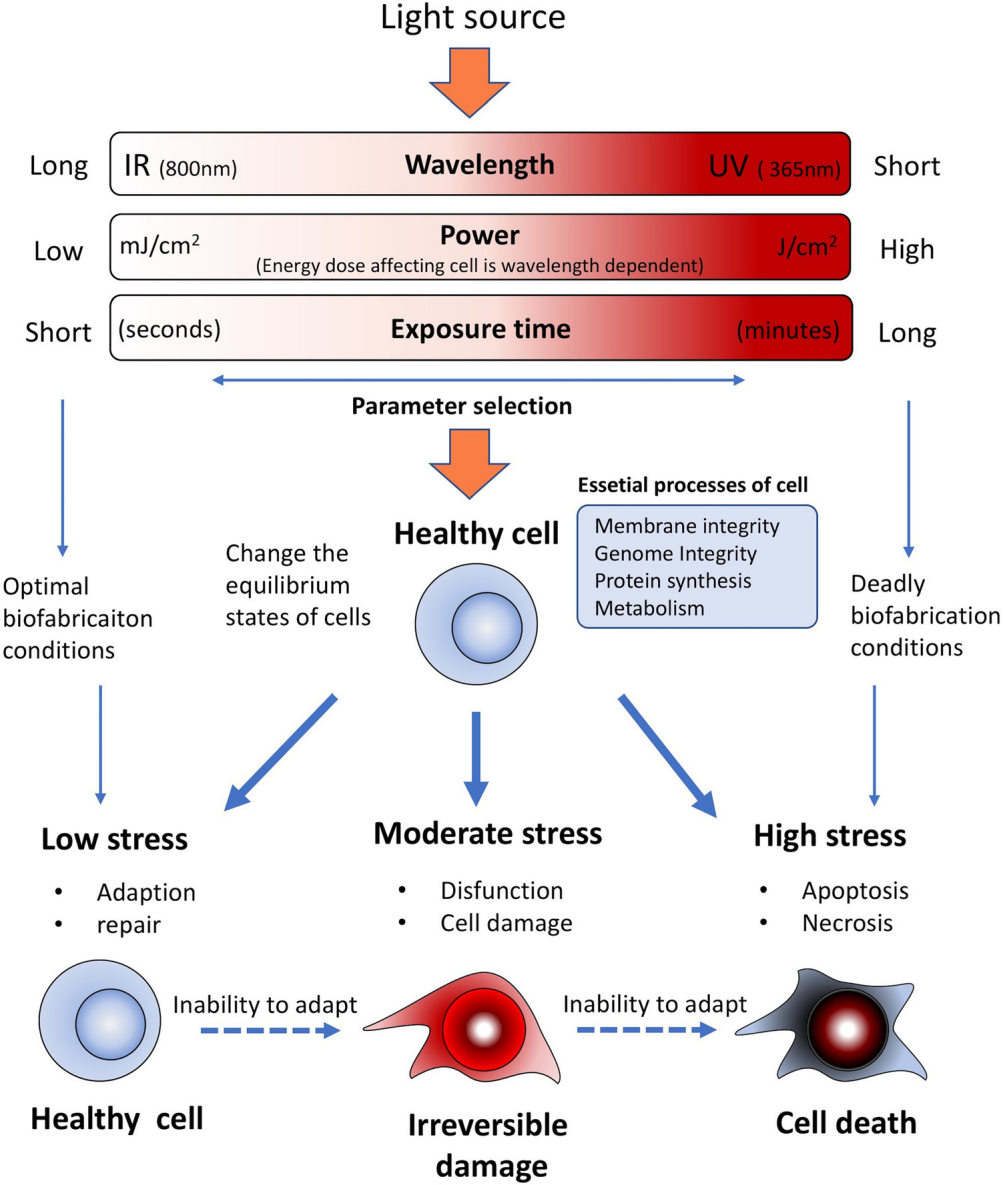
Diagram of the incident light parameters in relationship with cell viability.

During stereolithographic biofabrication, all the above-mentioned mechanisms need to be considered to increase cell viability. Light power, selected wavelengths, and exposure time are perhaps the main determinants during the biofabrication process (Fig. 5). Catros *et al.* have investigated the effect of laser energy on the viability of endothelial cells. They found that with a 1064 nm laser and energy configuration around 8 *μ*J, with a frequency of 5 KHz, cell damage was not induced, which if ascends to 24 *μ*J an increase in cell mortality can be expected.^73^ It is essential to adapt the light beam and optimize it to minimize cell damage without losing its ability to light cure or achieve cross-linking in the medium. The risk associated with the damage of cells during the TPP process needs to be considered prudently for efficient cell-laden biomaterial development. It was demonstrated that when using femtosecond laser pulses (790 nm, 1.5 W, 140 fs), the cytoskeleton of cells can be damaged. The energy of the pulse necessary for biomaterial photopolymerization and its modification by TPP is lower than the energy required for damaging the cytoskeleton structure due to laser ablation. Since TPP is a low thermal process, cells, proteins, and other agents can be added to the bioink without considering an important thermal damage associated with the bioprinting process, which in addition to preserving the DNA from being damaged is a critical point for its application in tissue engineering.

One of the main parameters affecting cell viability when using photosensible polymers is the use of UV light. During the photo-cross-linking progression, the PI absorbs the UV light and generates free radicals, which form the polymer network by polymerization.^74^ So, the light seems to be a critical parameter here; photons must have enough energy to induce the photopolymerization reaction, but remain below an energy damage threshold to avoid DNA damage or reactive oxygen species (ROS). For preserving cell viability, the quantum field (photons emitted in response to the light absorbing molecule) is important to consider, and the PI needs to preserve low irradiation energy for photopolymerization.^75,76^ Most PIs suitable for bioprinting, including Eosin Y and benzylidene cyclanone dyes, own high quantum yields for a low energy wavelength (∼800 nm). Salt-based PIs (LAP and Irgacure 2959) own high quantum yields with conversion kinetics very fast for a high energy wavelength (∼400 nm).^77^ Thus, cytocompatibility wavelength ranges usually are in the range of near-UV light (λ = 300–400 nm). In fact, using several photoinitiators has been proved to maintain high cell viability working at low concentrations with short UV exposure time and low intensity resulting in good cell viability. Miri *et al.* have used LAP in combination with poly(ethylene glycol) diacrylate (PEGDA) and gelatin methacryloyl (GelMA) at LAP concentrations of (1.0% w/v) and (0.03% w/v), respectively. Using a DMD bioprinter working at 365 nm, in combination with microfluidics, they were able to generate biological tissues structures such as tumor angiogenesis, muscle strips, and musculoskeletal junctions.^8^ However, large UV exposure times are usually required during 3D bioprinting of thick structures, which can diminish the cell viability or promote DNA damage of cells.^78^ Then, using the highest cytocompatible light intensity and optimizing the concentration of PIs for an efficient and fast cross-linking process in combination with a short light exposure time allow reducing prolonged cell exposure to free radicals.^79^

Recently, Ruskowitz and DeForest have demonstrated that the rate of proliferation remains the same and no cell death was observed when irradiating fibroblasts and human mesenchymal stem cells (NIH3T3) with a variety of light intensities (1, 5, 10, and 20 mW cm^−2^) at 365 nm. Nevertheless, they found that cells increase apoptosis by caspase activation in response to UV induced oxidative stress resulting from a UV light exposure (λ = 254 nm, 30 s at 300 *μ*W cm^−2^). A deeper analysis revealed that using a UV light of 365 nm does not alter the proteome nor shift protein production. Meanwhile, as an effect of induced DNA damage, 24 h after the light exposure at 254 nm wavelength, 40 proteins were differentially expressed including the down-regulation of several histones and the up-regulation of the cellular tumor protein p53. These results showed that light-induced cell death is wavelength-dependent, which emphasize that for 3D biofabrication, using photoresponsive biomaterials is a key factor to appropriately select light treatments.^80^

Bioprinting using living cells was demonstrated with primary cells, adult stem cells, and immortalized cell lines, with a variety of bioprinting technologies and biomaterials;^81^ and more recently with induced Pluripotent stem cells (iPSCs).^82^ Among these cell sources, iPSCs represent a huge potential in bioprinting for regenerative medicine, modeling diseases, and toxicological studies. Since the recent discovery of iPSCs and due to their unlimited self-renewal and pluripotent differentiation capabilities, they have been used for investigating disparate biological mechanisms.^83^ The indefinite division and pluripotency properties of iPSCs result in an unlimited source of any adult healthy and diseased cell type. However, iPSCs are more sensitive to handling procedures, which can influence pluripotency and differentiation. In this sense, although iPSCs are UV sensitive, the nature of the light based bioprinting process (noncontact process) represents the main advantage. Koch *et al.* have investigated laser bioprinting of undifferentiated human iPSCs in combination with different biomaterials and they have analyzed their impact on pluripotency and differentiation of cells. They found that hiPSCs are indeed more sensitive to the applied biomaterials, but not to laser printing itself.^84^ Additionally, iPSCs are also an attractive cell source that can avoid the ethical issues of embryonic cells.^85,86^ Embryonic stem cells (ESCs) are also of great interest for light-based bioprinting, nevertheless they are more sensitive to DNA damage in comparison with iPSCs. For example, mouse ESCs (mESCs) seemed to be more sensitive to UV or γ-ray irradiation than differentiated mouse embryonic fibroblasts (MEFs).^87^ Regarding DNA damage response (DDR), both human iPSCs and hESCs are analogous, showing high sensitivity to DNA damaging agents in comparison with somatic cells.^88^ For bioprinting, overcoming the DNA damage and cell viability related to the high UV sensitivity of iPSCs and ESCs requires novel biomaterials and photoinitiations in the visible range. This, would make it possible to investigate in the laboratory what is wrong in the diseased cells of an individual so that they give rise to the manifestation of the disease. These potentials place iPSCs in a privileged place to become, in the not too distant future, as an essential tool in 3D bioprinting.

## IV. FUTURE OUTLOOK AND CONCLUSIONS

Light–cell interactions are crucial parameters to consider in light-based bioprinting. Light properties determine the interaction mechanism for linear and nonlinear absorption. The light working parameters (power, exposure time, and repetition rate) have to be considered to improve cell viability and avoid DNA damage and ROS formation. Biomaterial thickness (representative in 3D bioprinting by the individual layer) increases cell survival: substrates with layer heights of 100 *μ*m showed higher cell viability compared to those of 20 *μ*m under equivalent light conditions. Biomaterial molecular weight optimization can promote cell viability. Small molecular weights may allow cell survival during the production process, but increase early death in the first few days. High molecular weights on the other hand may limit cell growth over time. Lasers and light sources with wavelengths in the visible spectrum range, appear to affect cell viability to a lesser extent (avoid damage induced by spectra such as UV), and should be of choice if technical manufacturing capabilities allow their use. Optimizing the porosity of the material to the targeted cell line would favor cell viability when exposed to the same light source. Temperature in the light or printing chamber should be controlled. Temperatures close to 30° during the manufacturing process will certainly improve cell viability. When we comment on absorption, we highlight how the concentration of the solute increased absorption in aqueous media. This must be key when choosing our bioinks, since they will not only affect the primary cell viability but will also be responsible for a greater or lesser absorption of light and consequently the possibility of cellular damage.

The suitable photopolymers for bioprinting are expected to have a high degree of conversion for minimizing the amount of residual monomer and pose fast photopolymerization kinetics to reduce the time of light exposure. An efficient photoinitiator should also have a broad range of photoactivity speed and power to reduce cell–light interaction time. UV light with wavelengths below 365 nm will cure the surface extremely quickly, but will damage cells. Using a wavelength of 385 nm or higher cures the material more uniformly with low cell damage and allows the light to penetrate and cure in thicker sections. The closer the wavelength to the visible range, the easier it is to bioprint using cells. Most of the PIs used in tissue engineering work within the UV wavelength range, which is indeed the major limitation because it has been demonstrated to be harmful to the cells and to the DMD array itself.^89^ Novel PIs working in the visible range are being developed and used for visible light photopolymerization of biocompatible polymers and are emerging as an optimal material for tissue bioprinting. Some researchers have adopted visible light stereolithographic approaches using such visible PIs. Tuan *et al.* have used a stereolithography system working in the visible range for polymerizing polyethylene glycol diacrylate (PEGDA) hydrogel containing cells with LAP.^13^ The LAP is still a UV-sensitive photoinitiator, though it can be cross-linked by a near-UV blue light. In the visible and near visible range, VA-086 (2,2′-azobis[2-methyl-n-(2-hydroxyethyl)propionamide]) which is sensitive at 385 nm (Ref. 52) and Eosin Y (2′,4′,5′,7′-tetrabromofluorescein disodium salt), which is sensitive at 514 nm can be included.^53^

Numerous innovations relating the use of TPP for biological applications, which envisage their unique opportunities for tissue engineering, have been described recently. TPP uses biomaterials and technologies that were initially developed for 3D printing. Therefore, in order to promote its bioprinting capabilities, an interdisciplinary approach is required to apply TPP technology for tissue engineering and biological applications. Ovsianikov *et al.* reported TPP hydrogel constructs containing cells^32^ where highly efficient two-photon photoinitiators and GelMA (gelatine-methacrylate) were used. MG63 cells (osteosarcoma cell lines) were encapsulated in Gel-MA; after laser exposure, cell damage was found in the laser-irradiated spot with a minor percentage of cells undamaged in the surroundings of the laser irradiated area. Experiments of Control (without photoinitiators) revealed that using the same laser parameters than those for TPP did not damage the cells. Conversely, cell damage seemed to be related to the cytotoxicity effects of some species, i.e., initiating radicals and ROS. These TPP features anticipate this technology as a suitable tool for biofabricating cell-laden 3D biomaterial constructs due to: (1) use of laser radiation near to IR (800 nm), which is able to penetrate deep into the hydrogels containing cells and avoid any cell damage; (2) TPP can be used under cell friendly conditions (pH and temperature); and (3) high water content hydrogels can be handled by 2PP. Urcciuolo *et al.* have recently demonstrated intravital 3D printing using cell-laden photosensitive photopolymer hydrogels within tissues of live mice at a wavelength of 850 nm.^90^ Intravital 3D bioprinting could serve as an *in vivo* alternative to conventional bioprinting which opens an interesting opportunity for three-photon polymerization (3PP) using longer wavelengths with the associated higher penetration depth.^91^

The aforementioned biophysical mechanism related to the light based bioprinting process can strongly influence the functionality, cell survival, DNA damage, long term cell viability, and phenotype maintenance of the bioprinted cell-laden structures. TPP is a promising 3D bioprinting process that uses harmless IR light on cells. So, cell laden bioinks are printed directly. Since tissue development involves specific chemical, physical, and geometrical environments to accomplish their anticipated functions, it would be required to design light sensible biomaterials with fitting biological properties, which will influence cell–light interactions. Although light based bioprinting can overcome the unresolved issues related to other bioprinting tools, such as shear stress and pressure, it must deal with the UV light toxicity for cells, which requires the development of novel bioinks and photoinitiators to move the working window more and more toward the visible light range.

## Data Availability

The data that support the findings of this study are available within the article.
